# Annual Report of the Postmaster-General

**Published:** 1892-02

**Authors:** 


Annual Report of the Postmaster-General of the United States, for the Year ending June 30th, 1891. Washington, Government Printing Office, 1891.

The Postmaster-General's annual report, just made public, shows that the usual annual deficit in the finances of the Department is gradually but surely disappearing. It amounted for the last year to only a little more than $6,000,000. The increase of the revenue last year, in spite of the loss on the transportation of lottery mail, is over $5,000,000.

The report describes in detail the plan by which promotions have been made during the year within the Department itself, and also recites how this general plan is to be extended to the whole service.

The Postmaster-General recommends this year the adaptation of the telephone, as well as the telegraph, to the postal system, showing that it is not only the constitutional privilege, but the duty of Congress to utilize all the means of modern science for quickening the transmission of intelligence.

Mr. Wanamaker believes in the full parcels post, but does not recommend it at present. He urges the abolition of personal suretyships of postmasters, as they are too frequently under obligations, which damage the service. He proposes to extend the money-order system everywhere.

Mr. Wanamaker describes the pneumatic-tube system of Berlin and London, and strongly recommends their adoption in this country. He insists that they would pay, and records several attempts at beginning such a service. A wonderful cancelling machine, which prevents the delay of letters in the post-offices in stamping, has been employed and a still more wonderful automatic electric stamper is in process of examination.

The Postmaster-General argues strenuously for chances to make deposits for people beyond the reach of savings banks.

